# C5-Predominant Segmental Zoster Paresis Presenting With Right Shoulder Abduction Weakness Before Cutaneous Eruption: A Case Report

**DOI:** 10.7759/cureus.116116

**Published:** 2026-09-11

**Authors:** Ryo Miyakawa, Yu Matsui, Tadamichi Shimizu

**Affiliations:** 1 Dermatology, University of Toyama, Toyama, JPN

**Keywords:** acute motor weakness, herpes zoster, magnetic resonance imaging (mri), segmental zoster paresis, varicella-zoster virus

## Abstract

Segmental zoster paresis (SZP) is an uncommon motor complication of herpes zoster (HZ) characterized by focal, asymmetric weakness in a myotomal distribution. Motor weakness usually follows the cutaneous eruption, and pre-eruptive weakness can mimic cervical radiculopathy, brachial plexopathy, neuralgic amyotrophy, or other causes of acute focal limb weakness. A 57-year-old woman developed right shoulder pain and, five days later, became unable to abduct her right arm. A vesicular eruption appeared on the lateral aspect of the right upper arm three days after the onset of weakness, and HZ was diagnosed. Examination showed C5 dermatomal vesicular lesions, severe pain, and right deltoid weakness of 2/5 on manual muscle testing. T2-weighted magnetic resonance imaging revealed hyperintensity and enlargement of the right C4 and C5 nerve roots. Nerve conduction studies of the median, radial, and ulnar nerves were normal; needle electromyography and axillary nerve conduction studies were not performed. The clinical and imaging findings supported a diagnosis of C5-predominant SZP, with localization most consistent with zoster-associated cervical radiculopathy. She received intravenous acyclovir and two courses of methylprednisolone pulse therapy. By hospital day 11, right deltoid strength had recovered to 5/5, and pain had decreased to 1/10. This case highlights the importance of considering pre-eruptive zoster-associated motor involvement in patients with acute painful focal limb weakness. Serial skin examinations and careful neurologic localization may facilitate timely diagnosis.

## Introduction

Herpes zoster (HZ) is caused by reactivation of varicella-zoster virus (VZV) latent in sensory ganglia and typically presents with acute neuropathic pain followed by a unilateral vesicular eruption in a dermatomal distribution [1]. Although sensory symptoms predominate, motor involvement can occur. Segmental zoster paresis (SZP) is an uncommon motor complication characterized by focal, asymmetric weakness in a myotomal distribution, usually corresponding to the affected dermatome, and occurs in approximately 0.3%-5% of patients with HZ [2].

Motor weakness usually develops after the cutaneous eruption; however, in rare cases, it may precede the rash [2-4]. When weakness occurs before typical skin lesions appear, the condition may initially resemble cervical radiculopathy, brachial plexopathy, or another cause of acute focal limb weakness [3-6]. We report a case of C5-predominant SZP in which right shoulder abduction weakness developed before the appearance of the cutaneous eruption.

## Case presentation

A 57-year-old woman with a history of glaucoma developed right shoulder pain without an apparent trigger. Five days later, she became unable to abduct her right arm. After another three days, a vesicular eruption appeared on the lateral aspect of the right upper arm. A local dermatologist diagnosed HZ and initiated oral valacyclovir 3,000 mg/day. Acetaminophen 2,000 mg/day and mirogabalin 10 mg/day were prescribed for pain. After the appearance of the vesicular eruption, she developed a headache one day before presentation to our department. On the following day, she was referred to our department for further evaluation. The headache was not accompanied by fever, neck stiffness, nausea, vomiting, altered consciousness, or other symptoms suggestive of meningitis or encephalitis.

On examination, vesicular lesions on an erythematous base were distributed over the lateral aspect of the right upper arm, clinically corresponding to the C5 dermatome (Figure 1). Right shoulder abduction was markedly impaired. Right deltoid strength was 2/5 on manual muscle testing (MMT), indicating full range of motion only with gravity eliminated. Pain intensity was 9/10 on the numerical rating scale (NRS). She was alert, with no cranial nerve abnormalities. Additional motor examination showed muscle strength of 3/5 in the right biceps, triceps, wrist extensors, and wrist flexors. The right biceps, triceps, and brachioradialis reflexes were absent, whereas the corresponding reflexes on the left were preserved. No superficial sensory loss was detected in the extremities or trunk.

**Figure 1:**
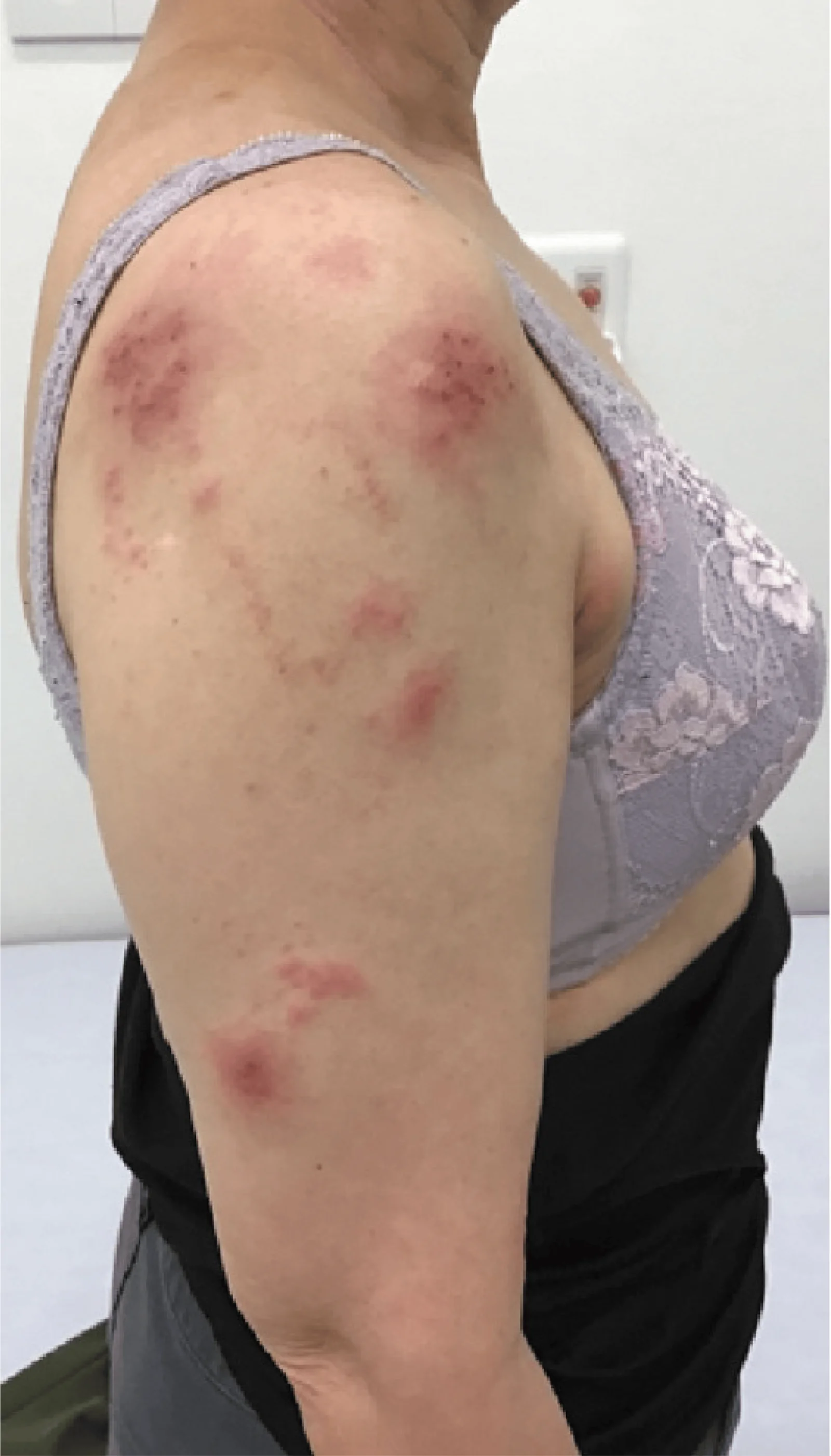
Cutaneous findings at initial presentation. Grouped vesicles on an erythematous base were present on the lateral aspect of the right upper arm, predominantly corresponding to the C5 dermatome.

Laboratory tests showed no leukocytosis or significant systemic inflammation, with a white blood cell count of 6,070/µL and a C-reactive protein level of 0.37 mg/dL. Serum VZV-specific IgM was negative, and VZV-specific IgG was positive. Given her headache, VZV-associated central nervous system involvement was considered in the differential diagnosis, and cerebrospinal fluid analysis was performed. The cell count was 8/µL, and the protein level was 21.1 mg/dL; a multiplex polymerase chain reaction panel including VZV was negative. Taken together, the clinical and cerebrospinal fluid findings were not strongly suggestive of meningitis or encephalitis.

T2-weighted magnetic resonance imaging (MRI) revealed hyperintensity and enlargement of the right C4 and C5 nerve roots (Figure 2). Nerve conduction studies of the median, radial, and ulnar nerves were normal and did not suggest diffuse peripheral neuropathy. Needle electromyography and axillary nerve conduction studies were not performed. Based on the C5 dermatomal eruption, predominant deltoid weakness, and right C4 and C5 nerve root abnormalities, we clinically diagnosed C5-predominant SZP with MRI findings suggestive of cervical nerve root involvement. The precise anatomical extent of the motor lesion was not definitively established.

**Figure 2:**
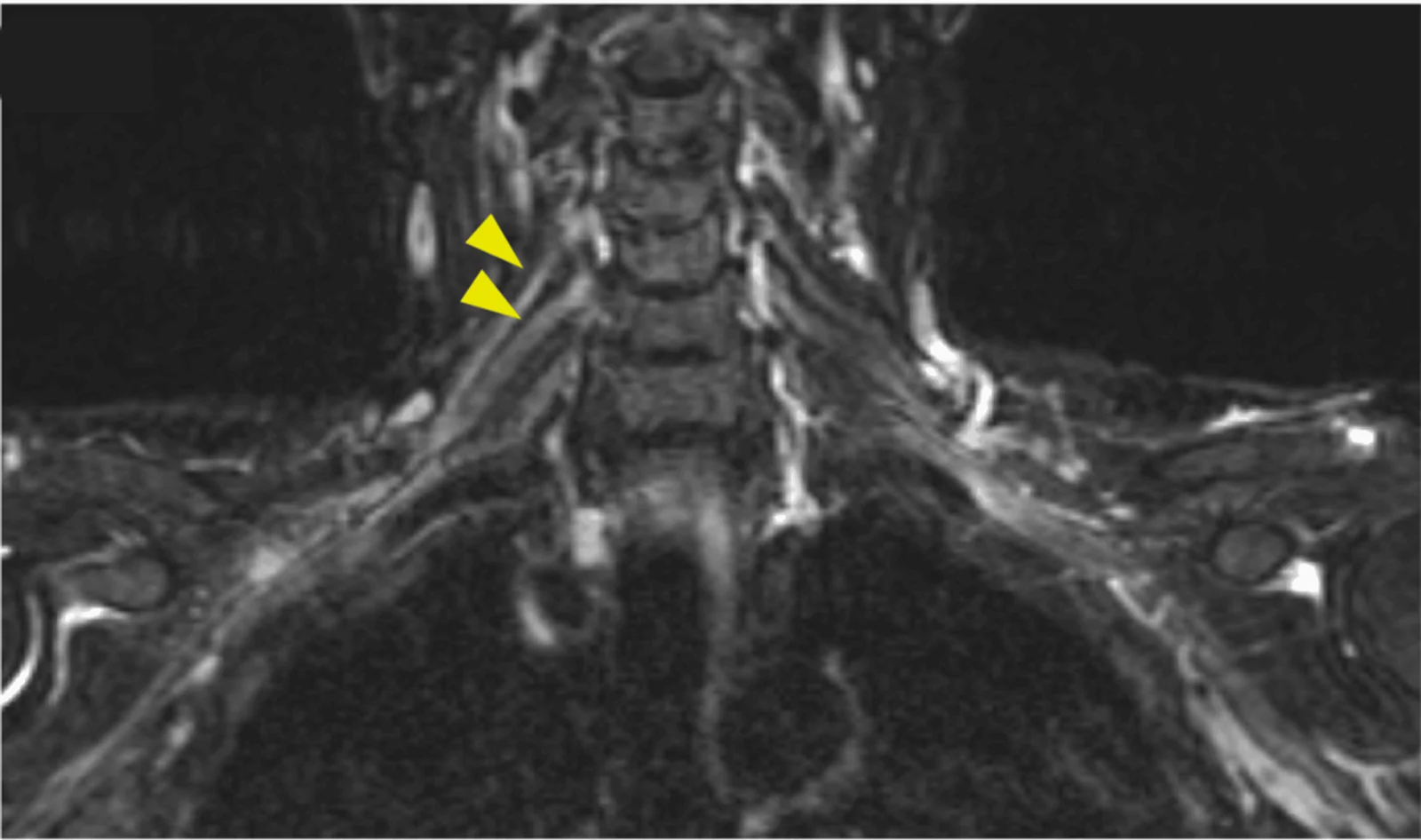
Cervical magnetic resonance imaging. Coronal T2-weighted imaging showed hyperintensity and enlargement of the right C4 and C5 nerve roots. Arrowheads indicate the right C4 and C5 nerve roots.

The patient received intravenous acyclovir 250 mg every eight hours for seven days. After consultation with the neurology department, two courses of intravenous methylprednisolone pulse therapy, each consisting of 1,000 mg/day for three consecutive days, were administered. The first course was given on hospital days two to four in the setting of severe focal weakness and MRI findings suggestive of nerve root inflammation. Right deltoid strength improved from 2/5 on admission to 3-/5 on hospital day five and 4-/5 on hospital day eight. With clinically significant deltoid weakness still present, a second course was administered on hospital days 9-11 as an individualized clinical decision. Right deltoid strength reached 5/5 on hospital day 10. On hospital day 11, strength remained 5/5 and pain intensity was 1/10 on the NRS. She was discharged on the same day.

At follow-up 26 days after admission, right deltoid strength remained 5/5. The right biceps and wrist extensors were also 5/5 on MMT, and the range of motion of the right shoulder was normal. Pain remained minimal at NRS 1/10. The patient reported a residual subjective sensation of heaviness in the right upper extremity, although a formal sensory examination was not documented at that visit. After this follow-up visit, care was transferred back to the referring hospital, and no subsequent neurological follow-up data were available to us.

## Discussion

SZP is an uncommon motor complication of HZ characterized by focal, asymmetric weakness in a myotomal distribution [2,7]. Motor weakness is thought to occur when zoster-associated inflammation extends to the anterior horn cells, ventral roots, nerve plexuses, or peripheral motor nerves [2,7,8]. Accordingly, neurologic involvement may occur at multiple levels, including the nerve roots, plexuses, and peripheral motor nerves.

A distinguishing feature of the present case was the combination of C5-predominant shoulder abduction weakness preceding the cutaneous eruption, MRI findings suggestive of cervical nerve root involvement, and rapid motor recovery. Before vesicular lesions appeared, this presentation could have been mistaken for degenerative cervical radiculopathy, brachial plexopathy, neuralgic amyotrophy, or another cause of acute focal limb weakness. The subsequent C5 dermatomal eruption, predominant deltoid weakness, and MRI abnormalities involving the right C4 and C5 nerve roots supported the clinical diagnosis of C5-predominant SZP with suspected cervical nerve root involvement.

Similar pre-eruptive motor weakness has been described in a small number of patients with HZ-associated limb paresis. The six previous reports identified in our targeted PubMed search described a total of eight patients with upper- or lower-limb involvement, in whom motor weakness preceded the cutaneous eruption by 3-10 days [3,4,9-12]. This targeted search was conducted to contextualize the present case and was not intended as a systematic review. Although the sites of neurologic involvement varied, these cases shared the unusual temporal pattern of motor weakness preceding the characteristic cutaneous eruption. These observations underscore the importance of serial skin examinations in patients presenting with acute focal limb weakness accompanied by pain. These cases and the present patient are summarized in Table 1.

**Table 1:** Previously reported cases of herpes zoster-associated motor paresis in which weakness preceded the characteristic cutaneous eruption, together with the present case. C = cervical; F = female; IV = intravenous; L = lumbar; M = male; MMT = manual muscle testing; MRI = magnetic resonance imaging; NRS = numerical rating scale; S = sacral

Reference	Age/Sex	Neurologic involvement	Interval from weakness to rash	Treatment	Outcome
Wendling et al. [3], Case 1	75/M	Right L5/S1 radiculopathy with foot drop involving the tibialis anterior and peroneal muscles	5 days	Valacyclovir 3,000 mg/day for 10 days	Residual motor weakness at 3 months
Wendling et al. [3], Case 2	80/F	Right L5 radiculopathy with ankle dorsiflexor weakness	10 days	Valacyclovir followed by IV and oral acyclovir; 20 days in total	Complete L5 paralysis (0/5) persisted at 3 months
Wendling et al. [3], Case 3	86/F	Right L5 radiculopathy with ankle dorsiflexor weakness	8 days	Valacyclovir 3,000 mg/day for 10 days; prior glucocorticoid therapy for severe pain (regimen not reported)	Complete motor recovery at 8 weeks
Tam et al. [4]	85/M	Left C4–C6 shoulder girdle weakness	3 days	Valacyclovir 3,000 mg/day for 7 days; physiotherapy and functional electrical stimulation	Incomplete motor recovery at 7 months; postherpetic neuralgia markedly improved
Çınar et al. [10]	59/M	Left C6–C7 radiculopathy with upper-limb weakness	5 days	Antiviral therapy; agent, dose, and duration not reported	Complete motor recovery; timing not reported
Umehara et al. [11]	72/M	Left C5 myotomal weakness; MRI abnormalities involving the spinal anterior and posterior horns and posterior root	10 days	Acyclovir 1,500 mg/day for 7 days; methylprednisolone 1,000 mg/day IV for 3 days	Residual motor weakness at 1 month
Dalugama et al. [12]	57/F	Left C5–C7 brachial plexopathy	Approximately 3–4 days	Acyclovir IV for 14 days; dexamethasone IV for 72 hours (dose not reported); gabapentin and physiotherapy	Complete motor recovery at 2 weeks after discharge
Rhyu et al. [9]	40/M	Left L2–L3 radiculopathy with lower-limb weakness	6 days	Valacyclovir 3,000 mg/day for 7 days	Complete motor recovery by 2 weeks after symptom onset; mild postherpetic neuralgia resolved by 2 months
Present case	57/F	Right C5-predominant upper-limb weakness, most pronounced in the deltoid	3 days	Valacyclovir 3,000 mg/day for 2 days, followed by acyclovir 250 mg IV every 8 hours for 7 days; methylprednisolone 1,000 mg/day IV for 3 days per course, administered in two courses (hospital days 2–4 and 9–11)	Complete motor recovery by 26 days after admission; deltoid strength recovered to MMT 5/5 by hospital day 10, with residual subjective arm heaviness

Accurate localization is important because the site and extent of zoster-associated limb paresis vary among patients [8]. Electrodiagnostic studies and MRI can provide complementary information for lesion localization. Electrodiagnostic studies may demonstrate axonal injury, and MRI may show nerve enlargement, T2 hyperintensity, contrast enhancement, or secondary denervation changes in affected muscles [5,7,8]. In our patient, nerve conduction studies of the median, radial, and ulnar nerves did not suggest diffuse peripheral neuropathy. However, needle electromyography and dedicated axillary nerve conduction studies were not performed; therefore, the clinically affected deltoid muscle and axillary nerve were not directly evaluated electrophysiologically, and the precise anatomic extent of the lesion could not be determined. The clinical deficit was C5-predominant, whereas MRI showed abnormalities of both the right C4 and C5 nerve roots. The clinical significance of the C4 root abnormality without clear clinical evidence of C4 involvement remains uncertain. Weakness in the triceps and wrist muscles, together with absent right upper-limb reflexes, also limited attribution of the deficits to an isolated C5 lesion. The combined clinical and imaging findings suggested cervical nerve root involvement, but concurrent plexus or peripheral nerve involvement could not be excluded.

Evidence guiding treatment for SZP remains limited. Treatment generally includes antiviral therapy, pain control, and rehabilitation [2,6]. Corticosteroids, including high-dose intravenous pulse therapy, have been used in reported cases, but evidence that they hasten motor recovery is insufficient [2,6]. Our patient improved after intravenous acyclovir and two courses of methylprednisolone pulse therapy; however, a causal treatment effect of corticosteroids cannot be inferred from a single case. The second pulse course was selected after persistent deltoid weakness following the first course. This was an individualized decision made with the neurology department, and neither the efficacy nor the optimal corticosteroid regimen for SZP has been established.

The prognosis of SZP is generally favorable, with approximately 67% of patients achieving complete or near-complete motor recovery in one literature review [2]. Recovery may nevertheless take several months, and residual weakness may persist [2,8]. In our patient, right deltoid strength reached 5/5 on hospital day 10 and remained 5/5 at follow-up 26 days after admission. This rapid recovery was clinically notable. However, no subsequent neurological follow-up data were available, limiting assessment of longer-term motor recovery, sensory symptoms, and neuropathic pain. Early recognition of this temporal pattern may help prevent misdiagnosis and unnecessary interventions while facilitating timely antiviral therapy, pain management, and rehabilitation.

## Conclusions

We report a case of C5-predominant SZP in which right shoulder abduction weakness developed before the appearance of the cutaneous eruption. The clinical presentation and course supported a diagnosis of C5-predominant SZP, while MRI suggested cervical nerve root involvement. Needle electromyography and dedicated axillary nerve conduction studies were not performed, limiting definitive anatomical localization. In patients with acute painful focal limb weakness, clinicians should consider pre-eruptive zoster-associated motor involvement, perform serial skin examinations, and undertake careful neurological localization.
